# Prevention of Abortion

**Published:** 1846-08

**Authors:** 


*Prevention of Abortion.*—The following practical remarks are from the pen of Dr. Meigs, the translator of Colombat: "I believe that the most successful mode of treating persons who are predisposed to abortion, before quickening, is one which I have long employed, and with a most satisfactory result. If the patient has had repeated miscarriages, I advise her to use an anodyne enema, consisting of a wine-glassful of boiled starch, mixed with forty drops of laudanum, to be taken at bed time, and to be repeated every night until quickening takes place. Perhaps the influence of the laudanum may be useful in suppressing or lessening the catamenial (?) act, or the efforts of the vesicular developments in the ovaries; or at least it may deprive the uterus of an abnormal degree of sensibility, the persistence of which might lead to its early contraction, and consequent expulsion of the fœtus."

